# The Role of Internal Limiting Membrane Flap for Highly Myopic Macular Hole Retinal Detachment: Improving the Closure Rate but Leading to Excessive Gliosis

**DOI:** 10.3389/fmed.2021.812693

**Published:** 2021-12-23

**Authors:** Yiqi Chen, Jun Wang, Xin Ye, Jiafeng Yu, Jiwei Tao, Li Lin, Sulan Wu, Jia Qu, Lijun Shen

**Affiliations:** School of Ophthalmology and Eye Hospital, Wenzhou Medical University, Wenzhou, China

**Keywords:** high myopia, inverted internal limiting membrane flap, macular hole retinal detachment, gliosis, spectral-domain optical coherence tomography

## Abstract

**Purpose:** To investigate the surgical outcomes of the inverted internal limiting membrane (ILM) flap technique for macular hole retinal detachment (MHRD) in high myopia.

**Methods:** This was a retrospective interventional study on 45 highly myopic eyes with MHRD after ILM peeling (*n* = 24, peeling group) or the inverted ILM flap technique (*n* = 21, inverted group). The inverted group was consisted of autologous blood (AB) subgroup (*n* = 10) and perfluorocarbon liquid (PFCL) subgroup (*n* = 11). MH closure, best-corrected visual acuity (BCVA), foveal microstructures, and excessive gliosis were analyzed during a follow-up of over 12 months.

**Results:** The inverted group achieved a higher MH closure rate (95.24%) than the peeling group (41.67%; *p* < 0.001). No significant difference was found in postoperative BCVA between the two groups (*p* = 0.98) or between the closed MH with or without excessive gliosis (*p* = 0.60). Excessive gliosis was identified in 9 eyes in the inverted group, and there was no difference in the incidence of excessive gliosis between the AB subgroup (4 in 10 eyes, 40%) and the PFCL subgroup (5 in 11 eyes, 45.45%) (*p* > 0.999).

**Conclusion:** The inverted ILM flap technique was effective in MH closure but had no advantage in postoperative BCVA and microstructural restoration. Excessive gliosis was only detected in the inverted group but did not affect the postoperative BCVA. Additionally, the incidence of excessive gliosis was not affected by adjuvants.

## Introduction

Macular hole retinal detachment (MHRD) is a severe vision-threatening complication of high myopia (1) and accounts for about 38% of the extreme myopia population in China (2). Although the underlying mechanism has not been delineated, it is currently accepted that MHRD is caused by the combined action of tangential traction and longitudinal force (3, 4). In highly myopic eyes, the tangential traction is associated with the attached cortical vitreous, epiretinal membrane (ERM), hardened internal limiting membrane (ILM), and retinal vessels (5, 6). With the posterior enlargement of the staphyloma and excessive elongation of the globe, longitudinal force plays a non-ignorable role in separating the neural retina from the retinal pigment epithelium (RPE) (3).

Patients with MHRD require immediate surgical intervention for vision rescue. The key to treating MHRD is releasing the tension entirely by removing the posterior hyaloid, ERM, and ILM. Although the retinal reattachment rate is high after the conventional pars plana vitrectomy (PPV) technique with membrane complex removal, the MH closure rate has been unsatisfactory, ranging from 25 to 86% (7–12). After introducing the inverted ILM flap technique, better anatomical success was achieved in recent years (8, 11–13). Previous studies have demonstrated that the inverted flap technique assisted by perfluorocarbon liquid (PFCL) can achieve a better functional and anatomical prognosis (8, 14). Besides PFCL, autologous blood (AB) containing rich growth factors has been applied in MHRD surgery to facilitate MH closure (15).

Although there have been several studies on the efficiency of the inverted flap technique vs. the ILM peeling technique, few previous studies have evaluated the speed of MH closure, the difference of foveal microstructural alterations between the two approaches, and the effectiveness of different intraoperative adjuvants (i.e., AB and PFCL) to date.

Thus, our study was designed to assess the postoperative recovery of eyes with MHRD undergoing different surgical approaches. To accomplish this, we compare the inverted ILM flap technique with the ILM peeling technique in terms of MH closure rate and speed, foveal microstructure, and visual outcomes. The postoperative results of AB-assisted and PFCL assisted-surgery were also compared in the meantime. Overall, our research can serve as a basis for better surgical design and more satisfactory surgical effects in the clinical treatment of MHRD.

## Materials and Methods

This retrospective case series study consisted of patients with MHRD who underwent ILM flap surgery assisted by AB or PFCL or traditional ILM peeling surgery from January 2015 to January 2020 at the Affiliated Eye Hospital of Wenzhou Medical University. The Institutional Review Board approved the study. Written informed consent was obtained from the subjects after they received a thorough explanation for the details of the study.

Inclusion criteria were as follows: (1) primary MH; (2) retinal detachment due to MH; (3) axial length longer than 26.5 mm; (4) follow-up period of 12 months or more. The exclusion criteria were as follows: (1) recurrent MH; (2) previous vitreoretinal surgery; (3) ocular diseases except for cataracts; (4) history of eye trauma.

**Figure 1:**
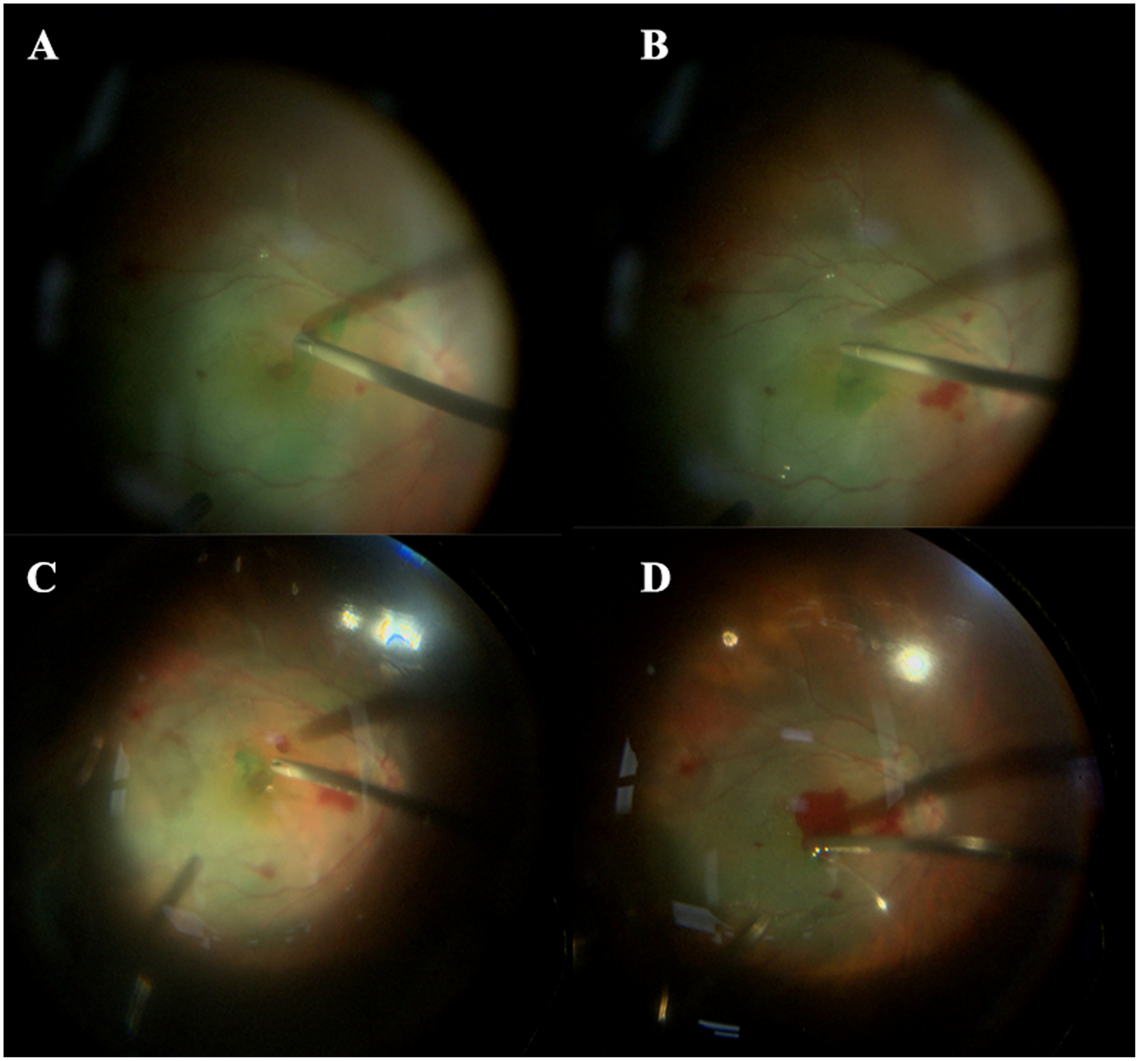
The surgical procedure for MHRD repair. Autologous blood-assisted inverted ILM flap surgery to treat a 60-year-old female patient with MHRD in her left eye. **(A)** ILM peeling: ILM is peeled off circularly for ~2–3 discs-diameters around the MH by ILM forceps. **(B)** The ILM flap remains attached to the edge of the MH; **(C)** the elevated ILM flap is inverted and covers the MH; **(D)** autologous blood is injected to cover the macula.

Two experienced vitreoretinal surgeons (SLJ and CYQ) performed all surgeries under combined retrobulbar and target-controlled infusion anesthesia. The PPVs were performed with a 23- or 25-gauge system (Accurus 800CS; Alcon Laboratories, Inc., Fort Worth, TX, USA). Phacoemulsification was performed in patients with cataracts, and an intraocular lens was implanted as needed. The surgical procedure was a standard three-port PPV with triamcinolone acetonide assisted posterior vitreous detachment (PVD), followed by removing the peripheral vitreous as much as possible. The ILM was stained with indocyanine green (0.025 mg/ml) for 5 s. In the peeling group, the ILM was peeled off circularly for ~2–3 discs-diameters around the MH by ILM forceps. In the inverted group, a modified inverted ILM flap technique assisted by perfluorocarbon liquid or autologous blood was performed to stabilize the ILM flap. In the PFCL subgroup, the nasal ILM was peeled off, and the free temporal ILM flap was hinged to the edge of the MH. Afterwards, subretinal fluid (SRF) was drained through the MH with a flute needle. Then, a small amount of PFCL was injected at the temporal side of the elevated flap to cover the MH. At the same time, the residual SRF was further removed through pre-existing peripheral retinal holes or tears. Subsequently, air-fluid exchange and air–PFCL exchange were performed. Consequently, silicone oil tamponade was applied. It is worth mentioning that the silicone oil was injected at the location of the optic disc to avoid washing away the ILM flap from the macula (Supplementary Video 1). In the AB subgroup, the nasal ILM was first completely peeled, and the temporal ILM was still attached to the retina. Then, the first air-fluid exchange was carried out for SRF drainage and temporary retinal reattachment, making the subsequent operations easier. After that, with vitreoperfusion restarting, the temporal ILM was not entirely removed, but a remnant was left attached to the edges of the MH. Next, we used the flute needle intentionally to drain the SRF through the MH and gently inverted the ILM flap to cover the MH. Small volumes of fresh blood from the patient were injected onto the macula, and then a blood clot was formed. Finally, a second air-fluid exchange was performed, and then silicone oil was injected as tamponade (Figure 1; Supplementary Video 2). Patients were required to maintain a prone position at least a week after surgery. Silicone oil removal was performed according to the postoperative recovery of the patient.

Information collected included demographic data such as age, sex, and laterality, pre-and postoperative BCVA, fundus photographs (CR-2, Canon, Japan), axial length (AL) (IOLMaster, Carl Zeiss, Germany), B-scan ultrasonography (Cinescan, Quantel Medical. Inc, Bozeman), and spectral-domain optical coherence tomography (SD-OCT; Spectralis HRA OCT; Heidelberg Engineering, Germany).

The postoperative parameters, including the rate and speed of MH closure, the pattern of MHRD recovery, foveal reconstitution, excessive gliosis, and retinal reattachment, were analyzed at 1, 3, 6, and 12 months after surgery and then yearly till the end of follow up. All the images were assessed by two retinal specialists independently.

MH closure was described as the disappearance of the foveal defect and no bare RPE exposed to the vitreous. Restoration of the external limiting membrane (ELM) or ellipsoid zone (EZ) was described as the corresponding continuous hyperreflective line in the fovea. Reconstitution of the outer nuclear layer (ONL) or inner nuclear layer (INL) was determined as the alignment of ONL or INL in the foveal area. Excessive gliosis has the following characteristics in OCT image: (1) a peculiar hyperreflective lesion representing a “peak-like” form; (2) extending from the RPE through the foveal defect and above the retinal surface; (3) the size of glial tissue increasing with time or remaining stable. Foveal dots of relatively higher reflectivity observed in the outer retinal layers were defined as hyperreflective foci (HF).

Statistical analyses were conducted with statistics software (IBM SPSS, version 26.0; IBM Corp., Armonk, NY, USA) to analyze the differences between the two groups or the two subgroups. Survival analysis was performed using R version 3.5.3, and MH closure or retinal reattachment was the end-point event. Before analysis, the decimal visual acuities were converted to the logarithm of the minimum angle of resolution (logMAR) units. The visual acuity of counting fingers, hand motions, and light perception were converted to 1.85, 2.3, and 2.6 logMAR units, respectively (16). The distribution of data was tested for normality with the Kolmogorov-Smirnov test. The student's *t*-test was utilized to compare normally distributed continuous data, whereas non-normal data were compared using the Mann-Whitney *U* test. Fisher's exact test was performed to compare the categorical data. Survival curves were assessed using the Log-rank test. A value of *p* < 0.05 was considered statistically significant.

## Results

In this study, 45 eyes of 45 patients were included. Twenty-four eyes were in the peeling group, and 21 were in the inverted group (AB subgroup: 10 eyes; PFCL subgroup: 11 eyes). The baseline characteristics are presented in Table 1, and no significant baseline differences were detected (Table 1). Notably, macular choroidal thinning was observed in all the eyes. Ten eyes (47.62%) of the inverted group and 11 (45.83%) of the peeling group were identified as Bruch membrane defects. At the last follow-up, the silicone oil of all patients had been removed from the vitreous cavity. No patients exhibited recurrence or required secondary surgery.

**Table 1:** Baseline characteristics of patients with MHRD who underwent ILM peeling technique or inverted ILM flap technique.

**Characteristics**	**Inverted group**					
	**Autologous blood**	**Perfluorocarbon liquid**	**Total**	***p***	**Peeling Group**	***P***
No. of eyes	10	11	21		24	
Age	58.3 ± 11.40	59.27 ± 10.33	58.81 ± 10.59	0.84*	55.42 ± 7.97	0.23*
Gender						
Male	0 (0.00%)	1 (9.10%)	1 (4.76%)	>0.999^†^	5 (20.83%)	0.19^†^
Female	10 (100.00%)	10 (90.90%)	20 (95.24%)		19 (79.17%)	
Axial length (mm)	29.45 ± 1.39	29.69 ± 2.00	29.59 ± 1.70	0.79*	29.92 ± 1.69	0.51*
Preoperative lens status						
Phakic	8 (80.00%)	10 (90.90%)	18 (85.70%)	>0.999^†^	20 (83.33%)	>0.999^†^
Pseudophakic	2 (20.00%)	1 (9.10%)	3 (14.30%)		4 (16.67%)	
Preoperative BCVA (logMAR unit, mean ± SD)	1.67 ± 0.35	1.87 ± 0.44	1.78 ± 0.40	0.43^‡^	1.62 ± 0.49	0.38^‡^
Presence of posterior staphyloma	8 (80.00%)	9 (81.82%)	17 (81.00%)	>0.999^†^	19 (79.17%)	>0.999^†^
Choroidal thinning	10 (100.00%)	11 (100.00%)	21 (100.00%)	>0.999^†^	25 (100.00%)	>0.999^†^
Bruch membrane defect	4 (40.00%)	6 (54.54%)	10 (47.62%)	0.67^†^	11 (45.83%)	>0.999^†^

*BCVA, best-corrected visual acuity; logMAR, logarithm of the minimum angle of resolution; SD, standard deviation*.

* *Student's t-test*.

† *Fisher's exact probability test*.

‡ *The Mann-Whitney U test*.

**Figure 2:**
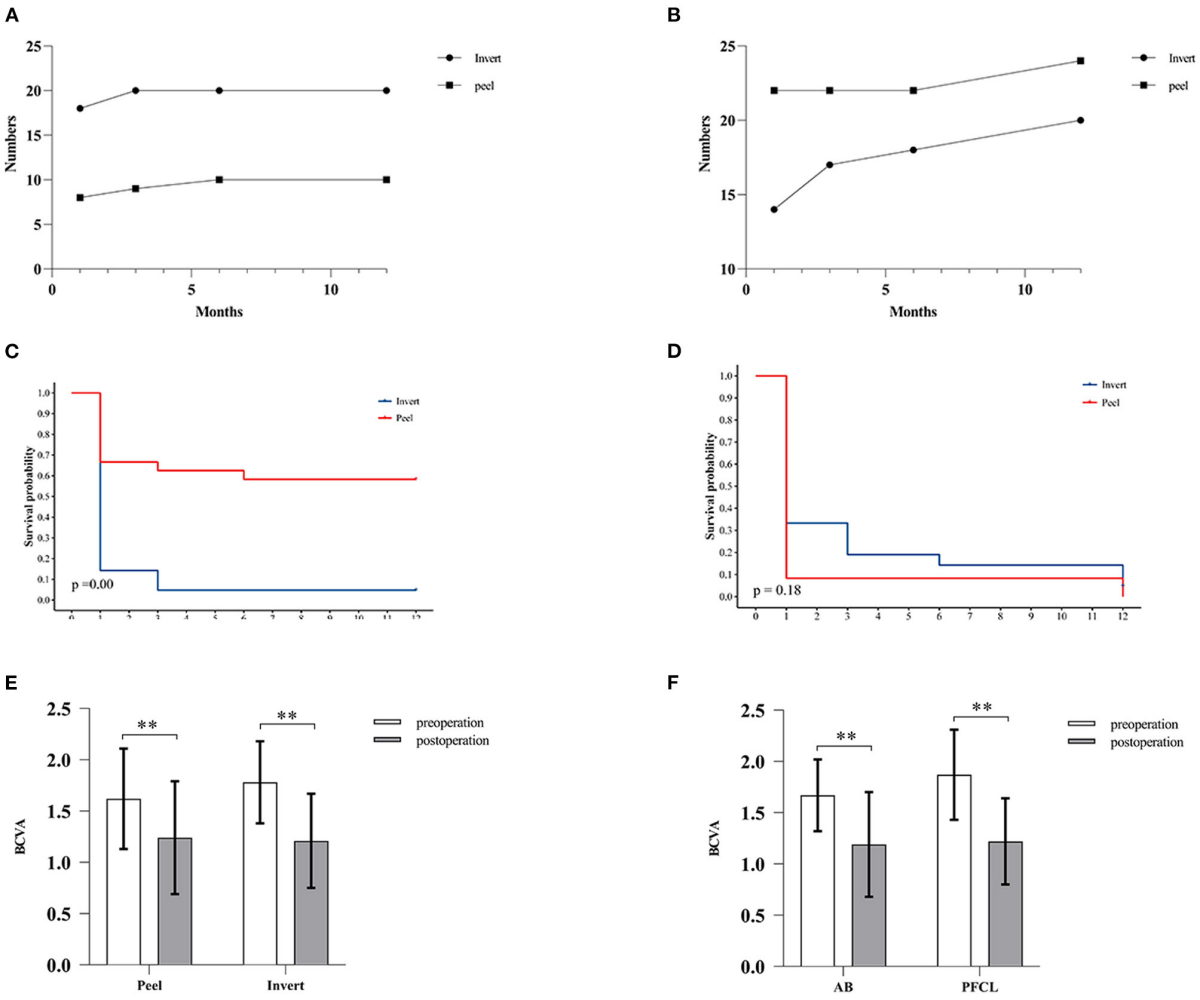
Comparison of MH closure, retinal attachment, and postoperative BCVA. **(A,B)** Graph showing the relationship between months and the number of MH closure cases **(A)** or retinal reattachment cases **(B)**. **(C,D)** Survival analysis. **(C)** The inverted ILM flap technique improved the MH closure rate. *Log-rank* test, *p* < 0.001. **(D)** No significant difference was found between the two groups in terms of retinal reattachment. *Log-rank* test, *p* > 0.05. **(E,F)** The bar charts showing BCVA at preoperative and postoperative visits. No significant difference in postoperative BCVA between the inverted and peeling group **(E)** or between the AB and PFCL subgroup **(F)** was identified. White histogram: preoperative BCVA; Gray histogram: postoperative BCVA. All the data are expressed as the mean ± SD.** *p* < 0.01, *Wilcoxon* test.

At 12-month follow-up, MH closure was found in 20 eyes (95.24%) in the inverted group, whereas only in 10 eyes (41.67%) in the peeling group (*p* < 0.001). No differences between AB and PFCL subgroups were found during the same follow-up period (Table 2). For the MH closure speed, 90% of MH closure in the inverted group (*n* = 18) and 80% of MH closure in the peeling group (*n* = 8) occurred during the first month after surgery (Figure 2). For the retinal reattachment, 20 eyes (95.24%) in the inverted group and 24 eyes (100%) in the peeling group got reattached (*p* = 0.47). All the eyes in the PFCL subgroup obtained retinal reattachment, while an eye assisted by AB was found without reattachment until the last follow-up (Table 2, Figure 2). During the first 3 months, the AB subgroup showed a slower rate of retinal reattachment in comparison with the PFCL subgroup (*p* = 0.04) (Table 2). No evidence of recurrent MH or detachment was identified.

**Table 2:** Postoperative anatomical and visual outcomes in patients with MHRD.

**Postoperative characteristics**	**Inverted group**					
	**Autologous blood**	**PFCL**	**Total**	***p***	**Peeling group**	***P***
No. of eyes	10	11	21		24	
Retinal reattachment (%)						
1 month	4 (40)	10 (91)	14 (67)	0.02^†^	22 (92)	0.06^†^
3 months	6 (60)	11 (100)	17 (81)	0.04^†^	22 (92)	0.40^†^
6 months	7 (70)	11 (100)	18 (86)	0.09^†^	22 (92)	0.65^†^
12 months	9 (90)	11 (100)	20 (95)	0.48^†^	24 (100)	0.47^†^
MH closure (%)						
1 month	8 (80)	10 (91)	18 (86)	0.59^†^	8 (33)	<0.001^†^
3 months	9 (90)	11 (100)	20 (95)	0.48^†^	9 (38)	<0.001^†^
6 months	9 (90)	11 (100)	20 (95)	0.48^†^	10 (42)	<0.001^†^
12 months	9 (90)	11 (100)	20 (95)	0.48^†^	10 (42)	<0.001^†^
Foveal microstructure (%)						
Excessive gliosis	4 (40)	5 (45)	9 (43)	>0.999^†^	0 (0)	<0.001^†^
INL restoration	1 (11)	1 (9)	2 (10)	>0.999^†^	2 (20)	0.58^†^
ONL restoration	1 (11)	2 (18)	3 (15)	>0.999^†^	1 (10)	>0.999^†^
ELM restoration	1 (11)	0 (0)	1 (5)	0.45^†^	0 (0)	>0.999^†^
EZ restoration	0 (0)	0 (0)	0 (0)	>0.999^†^	0 (0)	>0.999^†^
Hyperreflective foci	3 (30)	7 (64)	10 (48)	0.20^†^	12 (50)	>0.999^†^
Postoperative BCVA (logMAR unit, mean ± SD)	1.19 ± 0.51	1.22 ± 0.42	1.21 ± 0.46	0.81*	1.28 ± 0.59	0.84*
BCVA improvement ≥3 lines in ETDRS charts	7 (70)	7 (64)	14 (67)	>0.999^†^	12 (50)	0.37^†^
Stable or deteriorated BCVA (%)	3 (30)	2 (18)	5 (24)	0.66^†^	10 (40)	0.34^†^

*PFCL, perfluorocarbon liquid; MH, macular hole; INL, inner nuclear layer; ONL, outer nuclear layer; ELM, external limiting membrane; EZ, Ellipsoid zone; BCVA, best-corrected visual acuity; logMAR, logarithm of the minimum angle of resolution; SD, standard deviation*.

* *The Mann-Whitney U test*.

† *Fisher's exact probability test*.

**Figure 3:**
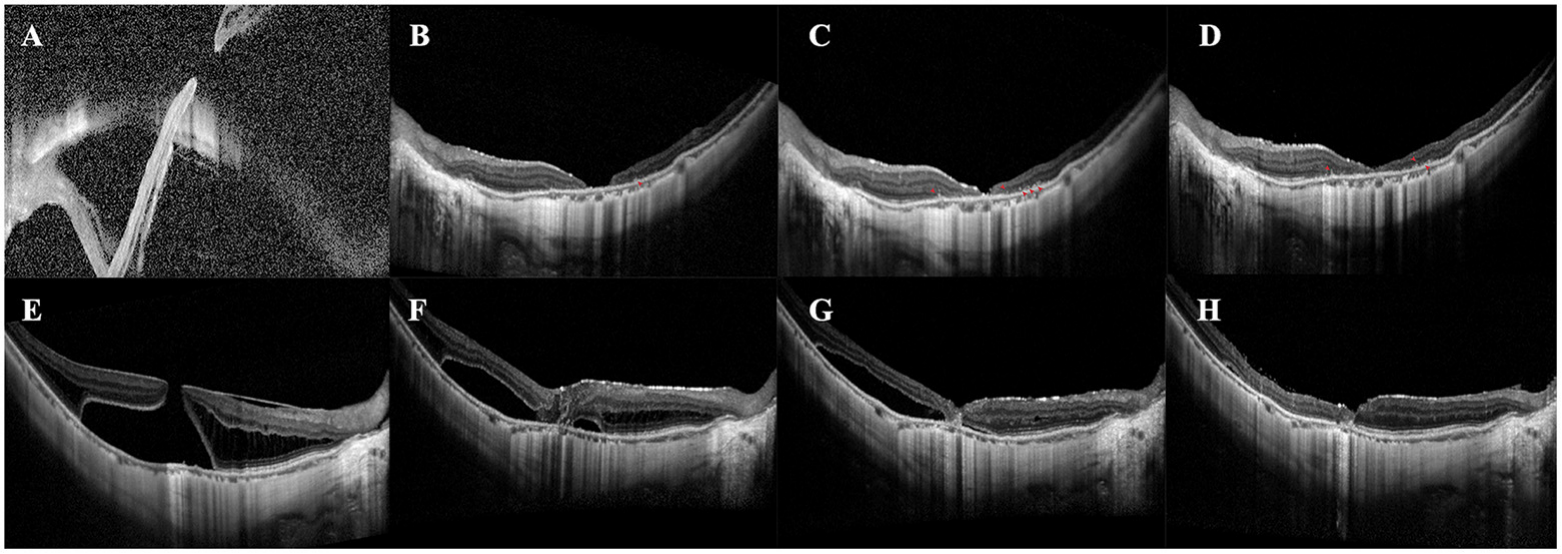
Two different patterns of MHRD recovery. **(A–D)** An example of Pattern 1. SD-OCT images of a 56-year-old woman receiving inverted ILM flap surgery assisted by PFCL. **(A)** The preoperative image shows MH and a detached retina. **(B)** Postoperative SD-OCT image at Day 7 shows the reattached retina with the foveal defect. **(C)** SD-OCT image at 2 months shows part of the foveal defect is filled with glial tissue. **(D)** Postoperative image at 3 months shows the foveal defect disappears and is replaced entirely by glial tissue. **(B–D)** Red arrowheads indicate hyperreflective foci located in the outer retinal layers. **(E–H)** An example of Pattern 2. SD-OCT images of a 49-year-old female patient receiving inverted ILM flap surgery assisted by autologous blood. **(E)** Preoperative SD-OCT image confirms the existence of MHRD. **(F)** Postoperative image at 1 month shows the MH is glued together with glial tissue and the retinal detachment still exists. **(G)** Postoperative image at 6 months shows the subretinal fluid is partially absorbed with uncomplete retinal reattachment. The volume of the glial tissue is significantly decreased. **(H)** Postoperative image at 8 months shows the MH closure with complete retinal reattachment. The retinal layers are disrupted, including the INL, ONL, ELM, and EZ.

Two distinct patterns of recovery were observed in the closed MH eyes. Pattern 1: The retinal reattachment occurred before MH closure. The foveal defect of the initial reattached retina was progressively filled by glial or normal tissue (Figures 3A–D). Pattern 2: The MH was firstly glued together by glial tissue. Then the retina reattached gradually with SRF absorption (Figures 3E–H). Thirteen eyes in the inverted group and 10 in the peeling group showed pattern 1 recovery. In comparison, seven eyes in the inverted group were presented with a pattern 2 recovery process.

In the inverted group, the mean preoperative BCVA was 1.78 ± 0.40, and the mean postoperative BCVA was 1.21 ± 0.46. The mean BCVA was 1.62 ± 0.49 preoperatively and 1.24 ± 0.55 postoperatively in the peeling group. Both groups showed significant improvements in their visual acuity after surgery. In addition, BCVA improved by more than 3 ETDRs lines in 14 eyes (66.67%) of the inverted group and 12 eyes (50.00%) of the peeling group, respectively. However, there was no significant difference in postoperative BCVA between both groups or subgroups (Table 2, Figure 2).

**Figure 4:**
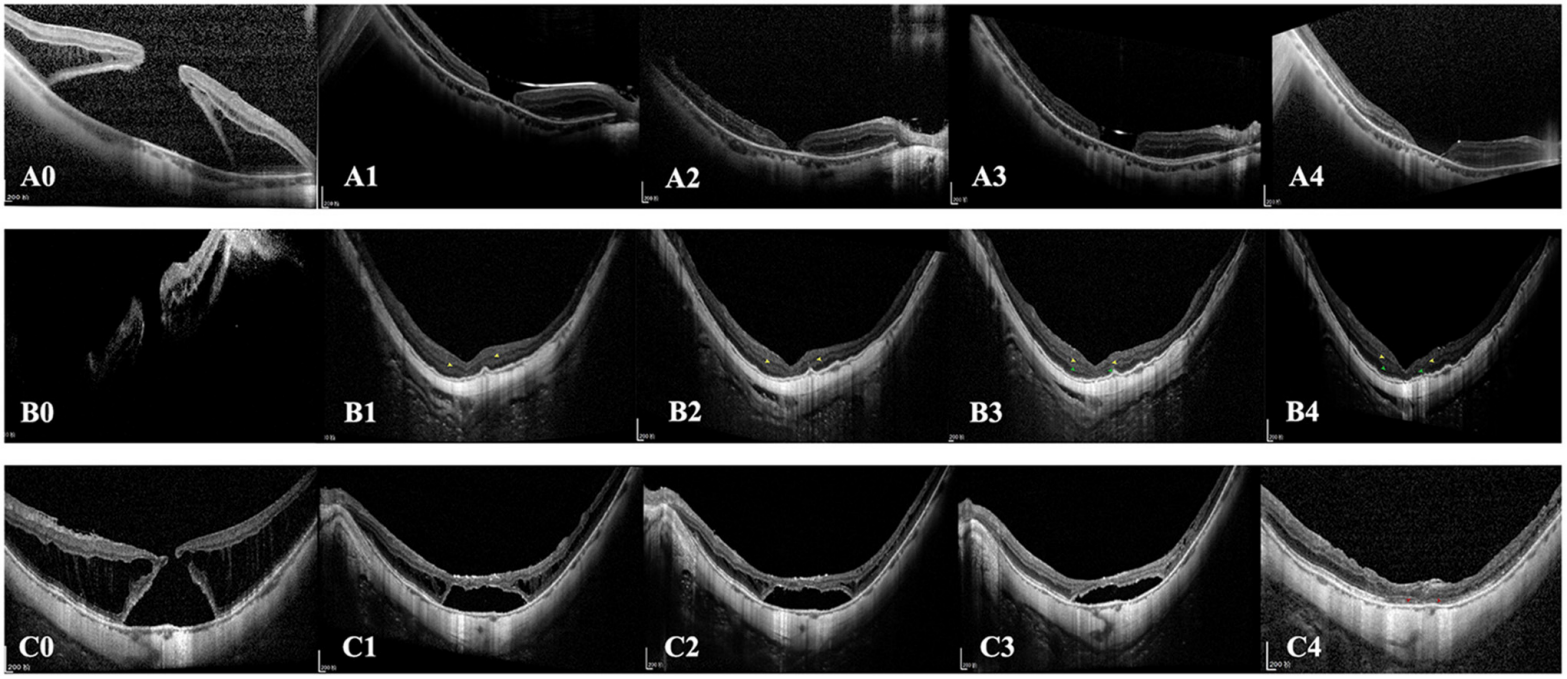
Cases of different postoperative outcomes and retinal layers reconstruction. **(A0–A4)** SD-OCT images of the left eye of a 48-year-old female patient with MHRD treated with the traditional ILM peeling technique. **(A0)** Preoperative OCT image shows the full-thickness MH and detached retina. **(A1)** 2-week postoperative SD-OCT image shows the retinal has reattached, and the foveal RPE is bare. **(A2)** 3-month postoperative, **(A3)** 6-month postoperative, and **(A4)** 12-month postoperative images show the MH is still unclosed. **(B0–B4)** SD-OCT images of a 62-year-old female patient with MHRD treated with perfluorocarbon liquid-assisted inverted ILM flap technique. **(B1)** 2-week postoperative and **(B2)** 3-month postoperative SD-OCT image shows the retinal has reattached and the MH is closed. The alignment of INL is observed at the foveal region (yellow arrowhead). **(B3)** 6-month postoperative and **(B4)** 12-month postoperative images show the restoration of INL and ONL at the fovea (green arrowhead). During the follow-up period, the outer hyperreflective layers are not visible in the whole macular region. **(C0–C4)** A 65-year-old male patient with MHRD treated with autologous blood-assisted inverted ILM flap technique. **(C0)** Preoperative status. **(C1)** 1-month postoperative, **(C2)** 3-month postoperative, **(C3)** 6-month postoperative, and **(C4)** 12-month postoperative SD-OCT images show the absorption course of subretinal fluid and the ELM reconstitution. Subretinal fluid is absorbed at the 12-month follow-up time-point, and retinal reattachment was eventually achieved. Notably, the ELM integrity is restored at 12-month follow-up (red arrowhead), and the EZ line is still interrupted.

**Figure 5:**
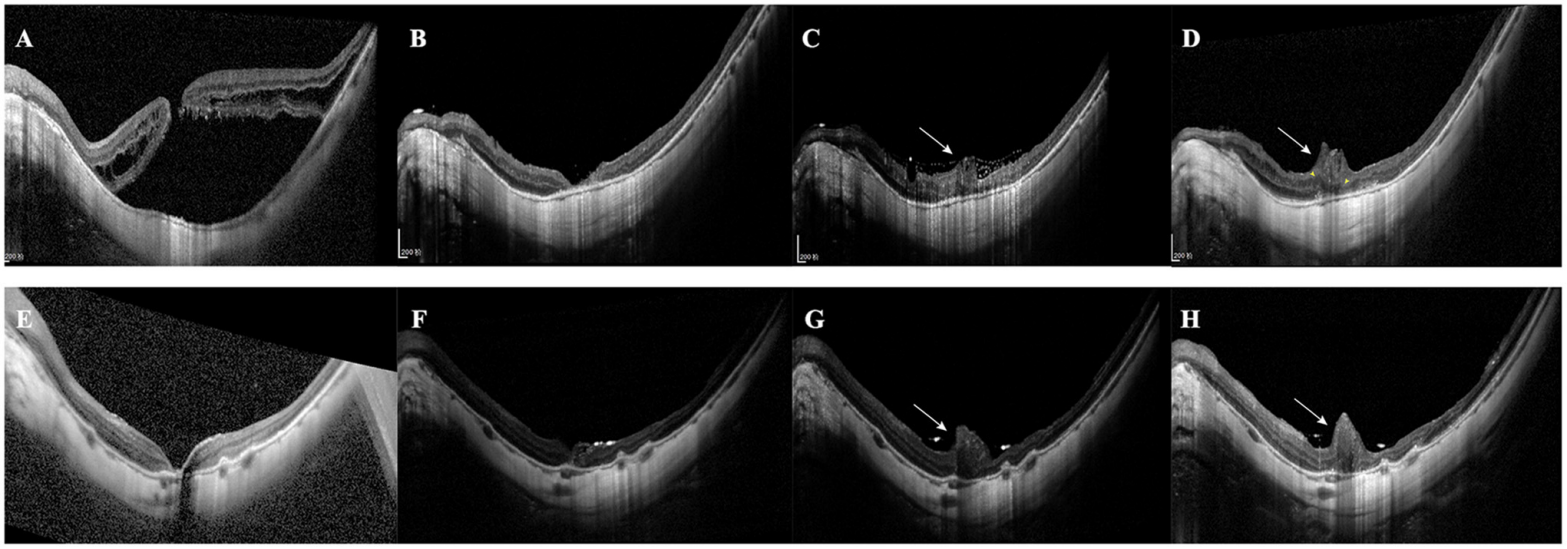
Excessive gliosis after the inverted ILM flap surgery. **(A–D)** A 68-year-old female patient with MHRD was treated with the autologous blood-assisted inverted ILM flap technique. **(A)** SD-OCT shows the MH and retinal detachment. **(B)** Postoperative 1st month. SD-OCT shows MH closure and retinal reattachment. **(C)** Postoperative 2nd month. SD-OCT shows the MH is filled with glial tissue. The tissue extends even beyond the retinal plane (white arrow). **(D)** 12-month postoperative SD-OCT image shows that the extent of excessive gliosis has increased (white arrow) and the alignment of INL (yellow arrowhead). **(E–H)** A 38-year-old woman with MHRD was treated with PFCL-assisted inverted ILM flap technique. **(E)** 1-week postoperative image shows the reattached retina with a minor foveal defect. **(F)** 1-month postoperative image shows the defect is filled with hyperreflective tissue and ILM flap is visible. **(G)** The 8-week postoperative image shows the volume of glial tissue remarkably increases (white arrow), and the ILM flap is still visible. **(H)** The 8-month postoperative image demonstrates a similar extent of excessive gliosis.

ELM (Figure 4) restoration was only observed in one eye of the inverted group. At the same time, INL (Figures 4, 5) and ONL (Figure 4) alignment were observed in 3 and 2 eyes, respectively. INL was observed in 1 eye in the peeling group, while neither ONL nor ELM restoration was presented. None of the eyes showed EZ reconstruction. Except for the partial restoration of regular retinal layers, 10 eyes of the inverted group and nine eyes of the peeling group presented HF (Table 2, Figure 3). Postoperative BCVA was compared between the closed MHs with or without HF. In the inverted group, patients with HF showed significantly better postoperative vision (0.99 ± 0.37) than without ones (1.41 ± 0.47) (*p* < 0.05). However, such a result was not obtained in the peeling group.

Excessive gliosis was detected in 9 eyes (42.86%) of the inverted group, while no similar lesion was observed in the peeling group (*p* < 0.001; Table 2, Figure 5). The lesion size increased slowly with time and tended to be stable (Figure 5). However, we did not detect significant differences when comparing the postoperative BCVA between the closed MH eyes with or without excessive gliosis (1.24 ± 0.46 vs. 1.13 ± 0.48; *p* = 0.60). Notably, excessive gliosis was observed in 4 of AB subgroup eyes (40.00%) and 5 of PFCL subgroup eyes (45.45%) (*p* > 0.999). It is worth mentioning that proliferative vitreoretinopathy was not observed in any of these eyes during the entire follow-up period, irrespective of the surgical approach.

## Discussion

MHRD as an intractable disease has been of great challenge for surgeons. The ILM peeling technique effectively reattached the retina but failed to achieve a satisfactory MH closure rate (7–12). Instead, the inverted ILM flap technique reached favorable anatomic results (8, 11–13). However, previous studies of the effect of these two techniques on the postoperative BCVA have shown inconsistent results. Takahashi et al. (11) and Sasaki et al. (13) investigated the surgical outcomes of MHRD eyes and found the inverted group had better postoperative BCVA than the peeling group. In contrast, several studies (8, 12, 17) pointed out that although the inverted flap technique effectively closed the MH, the BCVA after vitrectomy showed no significant superiority over the ILM peeling technique. These observations were in line with our data. Our inverted group's rate of MH closure was 95.24%, whereas only 41.67% in the peeling group. Both techniques achieved visual acuity improvement at the last follow-up, but there was no significant difference in the postoperative BCVA between the two surgical procedures.

Earlier studies have proven the recovery of ELM and EZ was correlated with better postoperative BCVA (18). In our study, only one eye showed the reconstitution of the ELM. At the same time, the restoration of the photoreceptor layer was prohibited by glial tissue in all other closed MH. Notably, around half of our patients showed BM defect preoperatively, which means the photoreceptor layer already occurred irreversible damage due to the extensive chorioretinal atrophy. In addition, the increase in visual acuity was partly due to retinal reattachment (19). It is interpretable that the postoperative BCVA of the two groups was similar. However, BCVA might not be comprehensive enough for assessing visual function. Microperimetry and multifocal electroretinography may be reliable tools for evaluating the macular function and offering information over BCVA (20, 21). Hence, these evaluation tools can likely be used in future researches.

**Figure 6:**
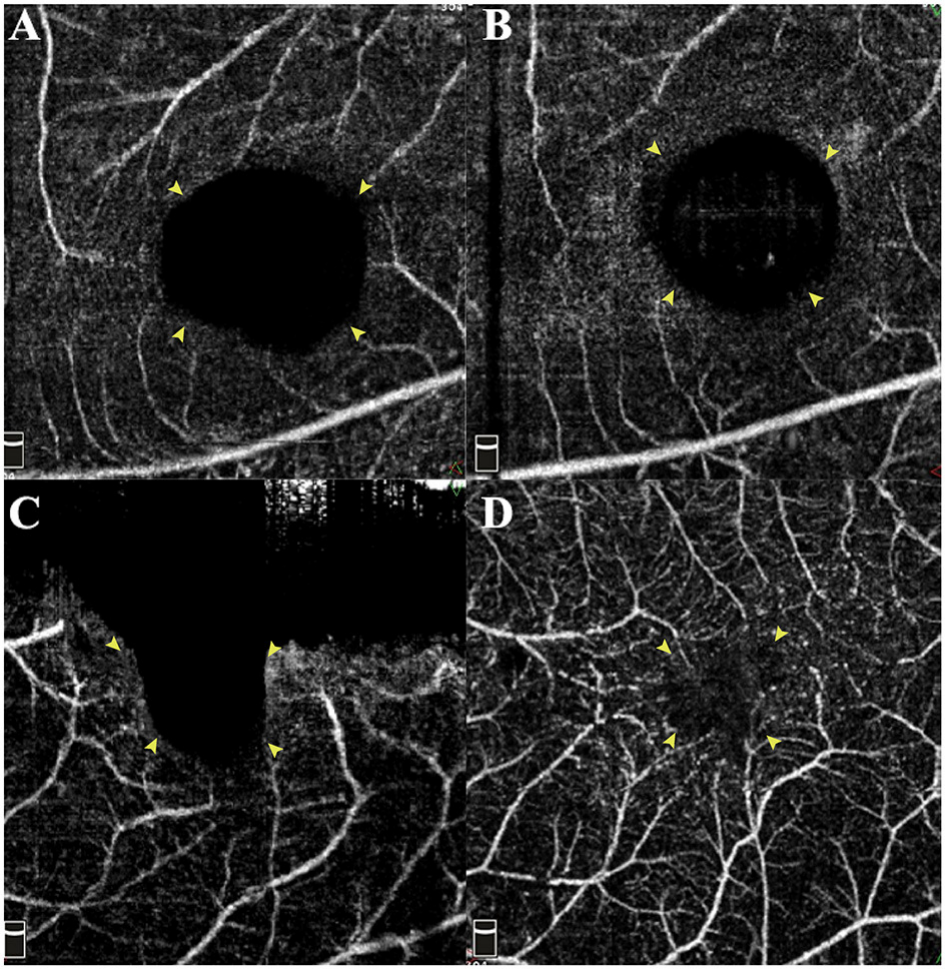
Changes of preoperative and postoperative superficial retinal capillary of the foveal region. **(A,C)** Preoperative macular OCTA images demonstrate the enlarged avascular region corresponding to the MH area. Compared with preoperative status **(A)**, the reattached macula with foveal defect shows no improvement in blood flow **(B)**. However, the wholly sealed MH **(D)** even by glial tissue contributes to the recovery of blood flow and reducing the non-perfusion area. Yellow arrowheads indicate the border of the avascular area.

There were two patterns of MHRD recovery in our patients. Pattern 1 was more common due to the relatively fast process of retinal reattachment, while the MH may need more time to close. This is most probably related to the activated glial cells spending time migrating. Pattern 2 occurred more frequently in the AB subgroup. This is because the flap plus AB clot formed a closed space that does not favor the drainage of residual SF through the MH. That was also why the AB subgroup showed a slower retinal reattachment rate. As reported by Bringmann et al., the regular hole closure is mediated by the concentric contraction of the outer plexiform layer (OPL) and the centripetal displacement of photoreceptors (22). After the foveal walls shift centripetally, the “Müller cell cone” remnants fuse and close the hole (22). If the remnants are absent or the central ONL contact RPE directly, the fovea will be filled with glial tissue (23). Simultaneously, the retraction of Müller cell outer processes that envelop the photoreceptor somata leads to the photoreceptors' centrifugal displacement. Hence, it could conceivably be hypothesized that our patients' “Müller cell cone” had been disrupted before surgery. Furthermore, already irreversible damage of the photoreceptor layer caused by atrophy may contribute to the direct contact of ONL and RPE. In this way, regular regeneration of the fovea is inhibited. However, the glial tissue also contributed to the recovery of blood perfusion and reduction of the avascular area. In contrast, there was no detectable blood flow signal of the macular capillary plexus in the “open” MH (Figure 6). Thus, the inverted group with more cases of closed MH exhibits its distinct advantages.

HF were only the observed phenomenon of this study, and the mechanism underlying this phenomenon was unclear, which was not the focus of our investigation. Our patients with HF showed better postoperative BCVA, contrary to the study reported by Hu et al. (24). Moysidis et al. investigated the anatomical and functional outcomes of autologous retinal transplantation to repair macular holes and MHRD. There was a high rate of HF noted in the graft in the early postoperative period. With the disappearance of the HF, the ELM and EZ band gradually reconstituted. Therefore, these HF suggest the presence of microglia, playing a role in wound healing (19). Kaya et al. guessed that HF are the deposition of ILM patches, glial cells and invaded RPE (25). In our study, the migration of the HF was observed. Thus, we boldly guessed that these foci might be cells of a particular type and participate in the repairing process. However, all these observations and speculations were based on OCT images, so the persuasive power was relatively limited. We'd like to vary our conjecture by further experiments in future work.

One unexpected finding was that excessive gliosis frequently occurred in the inverted group, while none of the eyes in the peeling group exhibited this phenomenon. The significant differences between the two groups reveal the role of the ILM flap, which may serve as the source of Müller cell fragments (8) and a “scaffold” to support the proliferation and migration of the Müller cells (26). The activated Müller cells may produce neurotrophic factors and basic fibroblast growth factors, which are also present in the flap (26). Gliosis is essential for closing MHs, but excessive gliosis may exert toxic effects on neurons. Although excessive gliosis did not affect postoperative BCVA, its impact on other aspects of visual function cannot be ruled out. A smooth, transparent plate is necessary to avoid image distortions (27), while the uneven retinal surface formed by excessive gliosis could cause metamorphopsia. Regrettably, assessment of visual distortion was not practiced in these patients. It is worth mentioning that the frequency of excessive gliosis did not differ significantly between the two subgroups. Although AB is rich in growth factors and cell-adhesion molecules, the ILM flap appears to serve a dominant role. Zhang et al. (28) co-cultured Müller cells with ILM and found the ILM promotes the proliferation of Müller cells. We speculated that high myopia might also play a specific role in this peculiar lesion. Several studies have reported that the ILM of pathological myopic eyes becomes severely thinner (29, 30). Additionally, Müller cells insert into the ILM as a possible response to the longitudinally elongate globe (29). These pathological alterations contribute to a significant increase of Müller cell debris remaining on the peeled ILM (29). Peeling the extremely thinning ILM are prone to cause severe mechanical injury and more cellular remnants of Müller cells adhering to the ILM, which creates a damage-repairing microenvironment conducive for glial proliferation.

All patients in this study were treated with PPV plus silicone oil tamponade. In cases of retinal detachment caused by MH in highly myopic eyes with extensive chorioretinal atrophy and posterior staphyloma, only using gas as an intraocular tamponade is insufficient. The loss of the choriocapillaris results in poor adhesion between the retina and underlying pigment epithelium, and the inverse traction caused by posterior staphyloma further reduces retinal adhesion (31). Therefore, silicone oil tamponade is recommended to enhance retinal-choroid adhesion and improve the initial retinal reattachment rate (32). Additionally, the high surface tension of silicone oil partially counteracts the stretching force around the MH in highly myopic eyes, which is conducive to MH closure (33). Furthermore, we used adjuvants to modify the inverted flap technique. The application of PFCL confers several advantages: (1) PFCL provides a good surgery view (34); (2) The flap can be grasped and inverted more easily with its surface tension (8, 34); (3) Its high specific gravity ensures the flap keeps stable and nearly complete drainage of SRF. Nevertheless, many drawbacks of PFCL, including toxic effects on cells, driving the higher likelihood of inflammations, and relatively high price, still need to be concerned. Comparatively, the complex of the AB and the inverted ILM plays an influential role as a macular plug and facilitates healing. In addition, various growth factors and components of fresh blood are known to be beneficial to MH closure (15). Although the application of AB may increase the difficulty of the surgery, the considerable effect and low cost determine its value as a superior alternative. Notably, the potential toxicity of autologous blood should not be ignored (15) and ought to consider in the future.

There are some limitations to be noted regarding the present study. First, the study was a retrospective design. Second, the sample size of our research is relatively small. Third, the relationship between anatomic findings and visual function was not explored. Some factors associated with visual prognosis, including the extent of retinal detachment, duration of disease, and MH size, need further investigation.

## Conclusion

In summary, the inverted ILM flap technique assisted by AB or PFCL is an effective treatment for MHRD, with a high success rate of MH closure. However, the application of this procedure is more liable to cause excessive gliosis unrelated to intraoperative adjuvants and had no significant advantage in terms of postoperative BCVA, MH closure speed, as well as foveal microstructure. Further prospective studies with longer follow-up time, diverse assessment of visual function, and quantified morphological indices of retinal anatomy are needed to determine the actual benefits and underlying mechanisms.

## Data Availability Statement

The raw data supporting the conclusions of this article will be made available by the authors, without undue reservation.

## Ethics Statement

The studies involving human participants were reviewed and approved by Research Ethics Committee of the Affiliated Eye Hospital of Wenzhou Medical University. The patients/participants provided their written informed consent to participate in this study. Written informed consent was obtained from the individual(s) for the publication of any potentially identifiable images or data included in this article.

## Author Contributions

LS had full access to all the data in the study and will take responsibility for the integrity of the data and the accuracy of the data analysis. YC and JW: study concept and design. JW, XY, and JY: acquisition, analysis, or interpretation of data. JW, XY, LL, and SW: drafting of the manuscript. YC and JT: critical revision of the manuscript for important intellectual content. JQ and LS: study supervision. All authors contributed to the article and approved the submitted version.

## Funding

The authors acknowledge the National Natural Science Foundation of China (81700884), Public Welfare Technology Research Project of Zhejiang Province (LGF21H12005), the Jointly Built Key projects of provincial and ministry of the National Health Commission (WKJ-ZJ-2037), and Wenzhou scientific research project (Y20190627). The sponsor or funding organization had no role in the design or conduct of this research.

## Conflict of Interest

The authors declare that the research was conducted in the absence of any commercial or financial relationships that could be construed as a potential conflict of interest.

## Publisher's Note

All claims expressed in this article are solely those of the authors and do not necessarily represent those of their affiliated organizations, or those of the publisher, the editors and the reviewers. Any product that may be evaluated in this article, or claim that may be made by its manufacturer, is not guaranteed or endorsed by the publisher.
